# *Aporosa tetragona* Tagane & V. S. Dang (Phyllanthaceae), a new species from Mt. Hon Ba, Vietnam

**DOI:** 10.3897/phytokeys.57.6347

**Published:** 2015-12-02

**Authors:** Shuichiro Tagane, Van Son Dang, Hironori Toyama, Akiyo Naiki, Hidetoshi Nagamasu, Tetsukazu Yahara, Hop Tran

**Affiliations:** 1 Center for Asian Conservation Ecology, Kyushu University, 744 Motooka, Fukuoka, 819-0395, Japan; 2 The VNM Herbarium, Institute of Tropical Biology, Vast, 85 Tran Quoc Toan Street, District 3, Ho Chi Minh City, Viet Nam; 3 Iriomote Station, Tropical Biosphere Research Center, University of the Ryukyus, 870 Uehara, Taketomi-cho, Yaeyama-gun, Okinawa, 907-1541 Japan; 4 The Kyoto University Museum, Kyoto University, Yoshida Honmachi, Sakyo-ku, Kyoto, 606-8501, Japan; 5 University of Science Ho Chi Minh City, 227 Nguyen Van Cu Street, District 5, Ho Chi Minh City, Viet Nam

**Keywords:** *Aporosa*, Hon Ba Nature Reserve, new species, Phyllanthaceae, Vietnam

## Abstract

A new species, *Aporosa tetragona* Tagane & V. S. Dang, **sp. nov.**, is described and illustrated from Mt. Hon Ba located in the Khanh Hoa Province, South Vietnam. This species is characterized by tetragonal pistillate flowers and fruits, which are clearly distinguishable from the other previously known species of the genus. The morphology and phylogeny based on *rbcL* and *matK* of this species indicated that the new species belongs to section Appendiculatae Pax & K. Hoffm.

## Introduction

The genus *Aporosa* Blume (1825) (Phyllanthaceae) comprises 82 species of small to medium sized trees distributed in various environments in South and Southeast Asia. They can be found in both primary and secondary forests, from lowland rain to dry deciduous, and as well as montane forest (up to 2200 m altitude in New Guinea) (Gagnepain 1927, Hô 2003, Schot 2004, Schot and van Welzen 2005). The genus is characterized by a dioecy, indumentum of simple hairs, petioles pulvinate at both base and apex, two glands on adaxial base of lamina (occasional), often with scattered disk-like glands on lower surface of lamina, axillary inflorescences, absence of petals, styles and disks, tiny staminate flowers with a minute or absent pistillode, and dehiscent regmata with persistent stigmas, sometimes beaked and/or stiped (Pax and Hoffmann 1922, Schot 2004).

The recent revision (Schot 2004) classified the genus *Aporosa* into five sections based on morphological analyses: sect. Aporosa, sect. Appendiculatae Pax & K. Hoffm., sect. Benthamianae Schot, sect. Papuanae Schot and sect. Sundanenses Schot. In Vietnam, two sections including 11 species with two varieties of *Aporosa* are recorded (Gagnepain 1927, Hô 2003, Schot 2004). Nine species with two varieties are included in Aporosa sect. Appendiculatae: *Aporosa ficifolia* Baill., *Aporosa macrophylla* Müll. Arg., Aporosa octandra var. octandra (Buch.-Ham. ex D. Don) A. R.Vickery (synonym: *Aporosa dioica* Müll. Arg. and *Aporosa oblonga* Müll. Arg.), Aporosa octandra var. malesiana Schot (synonym, *Aporosa microcalyx* (Hassk.) Hassk.), *Aporosa planchoniana* Baill., *Aporosa serrata* Gagnep., *Aporosa tetrapleura* Hance, *Aporosa villosa* Baill. (synonym: *Aporosa sphaerosperma* Gagnep.), *Aporosa wallichii* Hook. f. and *Aporosa yunnanensis* (Pax & K. Hoffm.) F. P. Metcalf. Two species are included in Aporosa sect. Sundanenses: *Aporosa duthieana* King ex Pax & K. Hoffm. and *Aporosa microstachya* Müll. Arg.

During a botanical survey of Mt. Hon Ba in Khanh Hoa Province, South Vietnam in 2014, an undescribed species of Aporosa sect. Appendiculatae was found at the margin of a broad-leaved evergreen forest near a stream, at 200–400 m altitude. Here, we describe and illustrate this plant as a new species, *Aporosa tetragona* Tagane & V. S. Dang.

In addition to the morphological examination, DNA sequences and phylogenetic analysis are extremely helpful for delimiting species (Hebert and Gregory 2005, Dick and Webb 2012). Here, we sequenced two DNA barcode regions, the partial genes for the large subunit ribulose-1,5-bisphosphate carboxylase oxygenase (*rbcL*) and maturase K (*matK*) (CBOL Plant Working Group 2009) to compare with related taxa.

## Materials and methods

### Morphological observations

The new species was recognized by detailed comparisons with morphologically similar species through literature review, dry specimens from the herbaria ANDA, BK, BKF, BO, HN, KYO, SING, TNS, VNM, and digitized plant specimens available on the web (e.g. JSTOR Global Plants (https://plants.jstor.org/)).

### DNA barcoding

Total DNA was extracted from silica-gel dried leaves collected in the field. DNA extraction was performed by a modified CTAB protocol (Doyle and Doyle 1987), as described in detail in Toyama et al. (2015). Amplification and sequencing of the two DNA barcodes regions, *rbcL* and *matK*, were performed according to published protocols (Kress et al. 2009, Dunning and Savolainen 2010).

### Phylogenetic analysis

In total, 22 accessions representing 14 species of *Aporosa* were included in phylogenetic analyses using DNA barcoding regions of *rbcL* (362 bp) and *matK* (392 bp) (Table 1). In addition to the new species, *Aporosa tetragona* Tagane & V. S. Dang, four species, *Aporosa aurea* Hook. f., *Aporosa microstachya* (Tul.) Müll. Arg., *Aporosa penangensis* (Ridl.) Airy Shaw and *Aporosa tetrapleura* Hance, were newly sequenced in the present study. The remaining sequences were obtained from GenBank. *Phyllanthus bokorensis* Tagane was used as an outgroup. The sequence alignment was performed by ClustalW with default parameter implemented in MEGA v 6.06 (Tamura et al. 2013).

**Table 1.** List of taxa used in this study with vouchers and GenBank accession number.

Section	Species	Vouchers	GenBank accession no.	
			*rbcL*	*matK*
Sect. *Aporosa*	*Aporosa frutescens* Blume	BT0095962054	KJ594599	KJ708827
Sect. *Appendiculatae*	*Aporosa aurea* Hook. f.	*Tagane et al. T4249*, FU	LC089033	LC089037
	*Aporosa ficifolia* Baill.	KYUM:5	AB925289	AB924682
	Aporosa octandra var. octandra (Buch.-Ham. ex D. Don) A. R.Vickery	SCBG007-1	KP094163	KP093256
	Aporosa octandra var. octandra (Buch.-Ham. ex D. Don) A. R.Vickery	SCBG007-2	KP094164	KP093257
	*Aporosa planchoniana* Baill. ex Müll. Arg.	KYUM:315	AB925549	AB924927
	*Aporosa planchoniana* Baill. ex Müll. Arg.	KYUM:945	AB925759	AB925129
	*Aporosa planchoniana* Baill. ex Müll. Arg.	KYUM:29	AB925313	AB924702
	*Aporosa tetrapleura* Hance	*Toyama et al. 1426*, FU	LC089030	LC089034
	*Aporosa tetragona* Tagane & V. S. Dang	*Tagane et al. V1976*, FU	LC050338	LC050339
	*Aporosa villosa* (Lindl.) Baill.	KYUM:994	AB925783	AB925152
	*Aporosa villosa* (Lindl.) Baill.	KYUM:127	AB925406	AB924795
	*Aporosa yunnanensis* (Pax & K.Hoffm.) F. P. Metcalf	J578	KR528750	KR530383
	*Aporosa yunnanensis* (Pax & K.Hoffm.) F. P. Metcalf	BB0195	KR528747	KR530380
	*Aporosa yunnanensis* (Pax & K.Hoffm.) F. P. Metcalf	G202	KR528748	KR530381
	*Aporosa yunnanensis* (Pax & K.Hoffm.) F. P. Metcalf	BB0194	KR528746	KR530379
Sect. *Benthamianae*	*Aporosa benthamiana* Hook. f.	BT0070230656	KJ594594	KJ708826
	*Aporosa lunata* (Miq.) Kurz	BT0070234186	KJ594600	KJ708829
Sect. *Sundanenses*	*Aporosa microstachya* (Tul.) Müll. Arg.	BT0070234330	KJ594602	KJ708830
	*Aporosa microstachya* (Tul.) Müll. Arg.	*Tagane et al. T4172*, FU	LC089032	LC089036
	*Aporosa penangensis* (Ridl.) Airy Shaw	*Tagane et al. T401*2, FU	LC089031	LC089035
Sect. *Papuanae*	*Aporosa papuana* Pax & K. Hoffm.	*Damas 004*, KYO	AB233915	AB233811
Outgroup	*Phyllanthus bokorensis* Tagane	*Toyama et al. 1740*, FU	AB936022	AB936023

The Neighbor-joining methods (Saitou and Nei 1987) with Maximum Composite Likelihood distance matrix (Tamura et al. 2004) implemented in MEGA v 6.06 was used to construct the phylogenic trees. Confidence values for individual branches were determined by bootstrap analysis with 10,000 repeated samplings of the data.

## Results and discussion

The new species belongs to the section Appendiculatae as the leaf lamina has basal adaxial glands (Fig. 2C), disc-like glands unevenly scattered within the arches of the marginal veins throughout the abaxial surface of the lamina (Fig. 2B), papillate stigmas (Fig. 2D), and pubescent septae and column in the ovary (Fig. 2E) (Schot 2004), but is distinguished from previously known species by its tetragonal ovary of the pistillate flower and the fruit.

**Figure 1.**
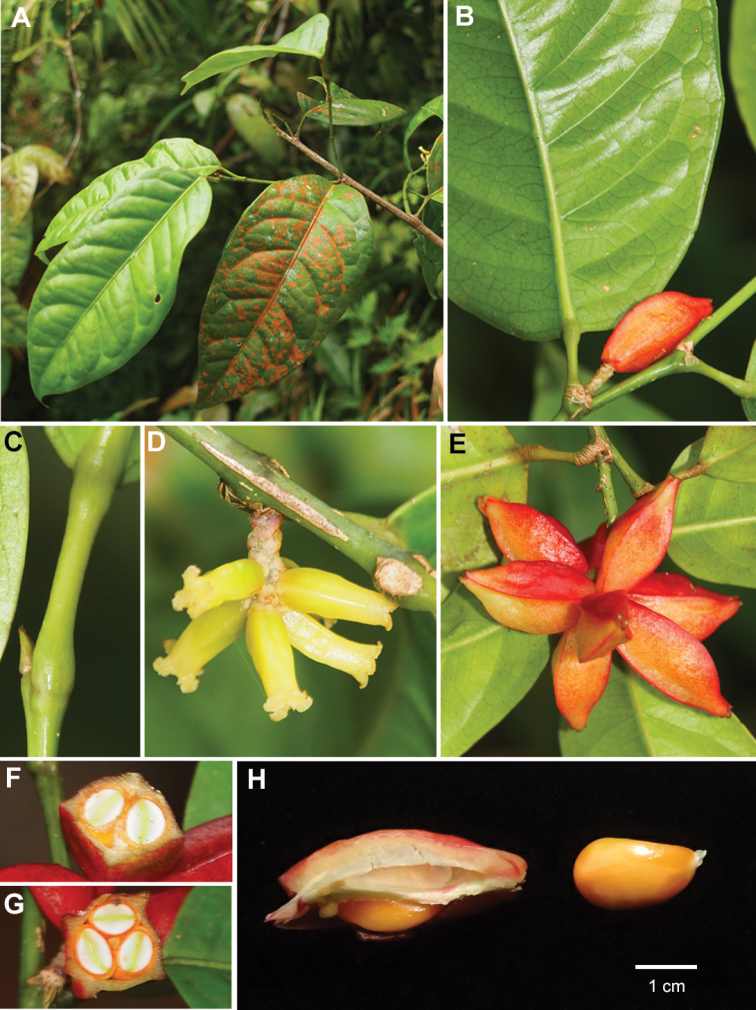
*Aporosa tetragona* sp. nov. **A** Leafy branch **B** Fruits and portion of abaxial surface of young leave **C** Apical bud and pulvinate petiole at both base and apex **D** Pistillate inflorescence **E** Fruits **F, G** Transverse section of fruits **H** Seeds taken from fruits. Materials: *Toyama* et al. *V1976*.

**Figure 2.**
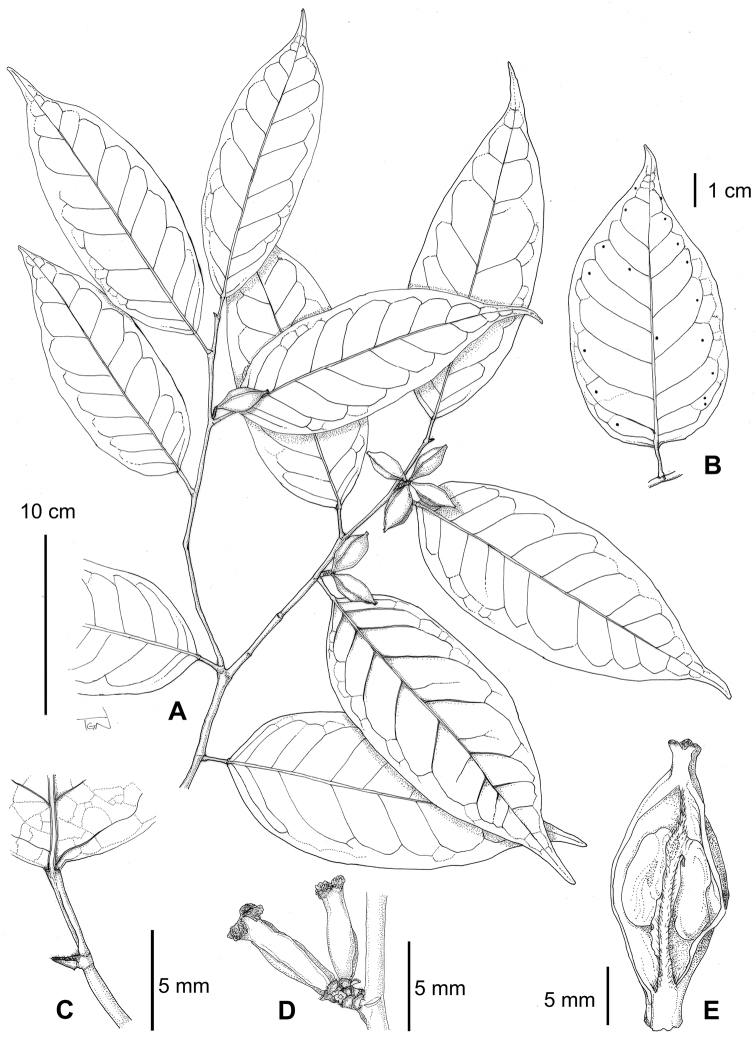
*Aporosa tetragona* sp. nov. **A** Fruiting branch **B** Schematic of the placement of the disc-like glands on the lower side of the leaf **C** Apex of branch **D** Pistillate inflorescence **E** Longitudinal section of fruits. Materials: **A**–**C**, **E** from *Toyama* et al. *V1976* (KYO), **D** from *Toyama* et al. *V829* (FU).

**Figure 3.**
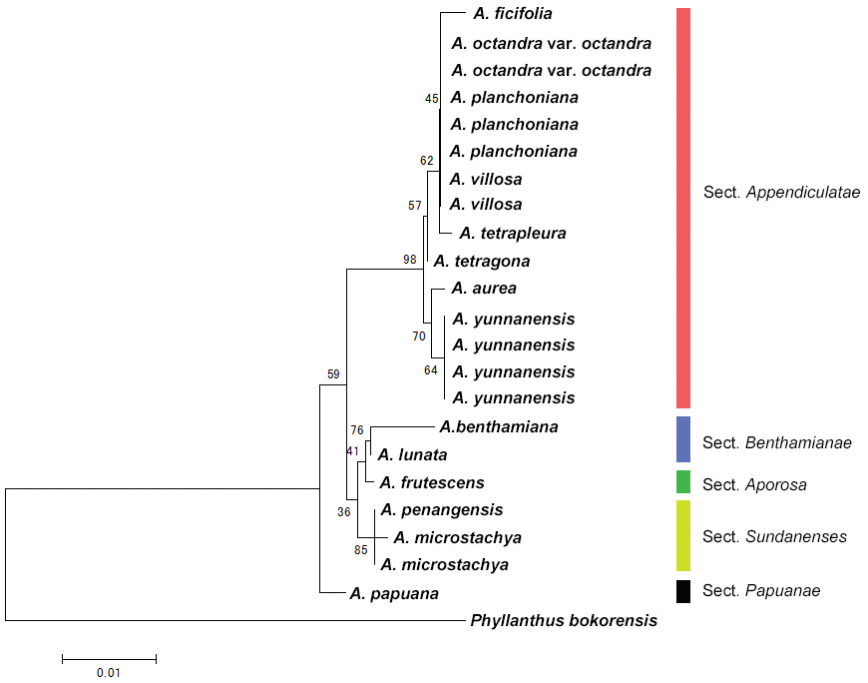
NJ tree of *Aporosa* species based on *rbcL* and *matK*. Branches are labeled with bootstrap support (% of 10,000 replicates).

The Neighbor-joining tree based on *rbcL* and *matK* supports the separation of each morphologically defined section and the monophyly of sect. Appendiculatae, sect. Benthamianae and sect. Sundanenses with 98 %, 76 % and 85 % bootstrap probability, respectively (Fig. 3). The new species was placed in sect. Appendiculatae and clearly separated from other species of this section with a sister relationship to the clade including *Aporosa ficifolia*, Aporosa odctandra var. octandra, *Aporosa planchoniana* and *Aporosa villosa*.

Thus, the species is morphologically distinct from known taxa and the phylogeny supports the separation from related species. Here, we define the new species *Aporosa tetragona* Tagane & V. S. Dang.

## Taxonomy

### Aporosa tetragona

Taxon classificationPlantaeMalpighialesPhyllanthaceae

Tagane & V. S. Dang sp. nov.

urn:lsid:ipni.org:names:77151272-1

Figs 1 , 2

#### Diagnosis.

*Aporosa tetragona* is distinct from all other *Aporosa* species by having a tetragonal ovary and fruit. The leaves are similar to *Aporosa acuminata* Thwaites, but differing in not only fruit shape but also wider leaves (vs. 2–4 cm wide), larger pistillate flowers (vs. 2–3.5 mm long), and glabrous fruits (vs. sparsely puberulous).

#### Type.

VIETNAM. Khanh Hoa Province, Mt. Hon Ba, edge of evergreen forest near stream, 12°06'30.60"N, 108°59'15.70"E, alt. 393 m, 22 November 2014, with female fl. and fr., *Toyama H., Tagane S., Dang. V. S., Nagamasu H., Naiki A., Tran H., Yang C. J. V1976* (holotype KYO!, isotypes BKF!, FU!, K, NTU!, P, VNM!, the herbarium of Hon Ba Nature Reserve!).

#### Description.

Small tree, 3 m tall. Twigs glabrous, young branchlets green *in vivo*, dull yellowish green to pale yellow *in sicco*, old branchlets light grayish brown. Stipules caducous, not seen. Leaves: petiole 0.8–1.7 cm long, sunken above, rounded below, pulvini distinct, glabrous; blade ovate to elliptic, (6.8–)9–16.5 × 3.9–7.0 cm, length/width ratio 2.0–2.9, chartaceous to subcoriaceus, completely glabrous, dull yellowish green to dull pale yellow above and beneath *in sicco*, base cuneate to rounded, or shallowly subcordate, basal glands present, margin entire, foliar glands abaxially scattered mostly within the arches of the marginal veins, apex acuminate, acumen up to 2.3 mm long; midrib prominent on both surfaces, or rarely sunken only on the upper surface, secondary veins 10–14 pairs, raised on the lower surface, tertiary veins reticulate, visible on both surfaces of young leaves *in sicco*, inconspicuous on lower surface of old leaves. Staminate inflorescences not seen. Pistillate inflorescences in axils of leaves near the top of branchlets, solitary, flowers up to 7, rachis 2–5 mm long, densely pubescent; bracts broadly triangular, ca. 1 × 1.1 mm, margin ciliate, very sparsely pubescent outside, glabrous inside. Pistillate flowers (6–)8–10 mm long, (1.8–)2.5–3 mm in diam., sessile, yellowish *in vivo*, reddish brown *in sicco*; sepals 4, triangular, 0.8–1.1 × 1.1 mm, glabrous to very sparsely hairy outside, glabrous inside except near base, margin ciliate; ovary obclavate, 5–9 mm long, tetragonal, 2-locular, glabrous outside; ovules 2 per locule; stigmas slightly raised, elongated, ascending from the top of the ovary, stigma bilobed, lobes ca. 0.6–1 mm long, each stigma lobe apically deeply bifid, papillate and hairless above, smooth and very sparsely hairy beneath, style remnant present. Fruits tetragonal ellipsoid with sharp ridges, 21–25 × 7–9 mm, stiped, beaked, fleshy, reddish *in vivo*, pinkish orange to reddish brown *in sicco*, glabrous; septae and column pubescent with hairs of 0.4–0.6 mm long. Seeds 2 or 3, ellipsoid, flattened, ca. 9.0 × 5.0 × 3.5–4 mm, covered by fleshy, yellow aril *in vivo*, yellowish brown *in sicco*.

#### Other specimen examined.

Vietnam. Khanh Hoa Province, Mt. Hon Ba, in evergreen forest near river, 12°06'33.41"N, 108°59'24.89"E, alt. 367 m, 19 Feb. 2014, with female fl., *Toyama* et al. *V829* (FU!, VNM!, the herbarium of Hon Ba Nature Reserve!).

#### Phenology.

Flowering specimens were collected in July and November; fruiting in November.

#### Distribution and habitat.

This species is currently known only from Hon Ba Nature Reserve, Khanh Hoa Province, South Vietnam. The small populations were found at the edge of humid broad-leaved evergreen forest close to a stream, altitude 200–400 m.

#### Etymology.

The specific epithet *tetragona* reflects the quadrangular shape of the ovaries in the pistillate flowers and fruits.

#### GenBank accession No.

*Toyama* et al. *V1976*: LC050338 (*rbcL*), LC050339 (*matK*).

#### Conservation status.

The species is known only from the type locality in Mt. Hon Ba at 200–400 m altitude. It is suggested that *Aporosa tetragona* should be placed under the IUCN category ‘Critically Endangered’ (CR) (IUCN 2012) because of its limited distribution with an area of occupancy estimated to be less than 10 km^2^ (criterion B2 a) and a small number of individuals estimated to be less than 250. Recent botanical inventories carried out in this narrow area along stream discovered several new species, including *Dillenia tetrapetala* Joongku Lee, T. B. Tran & R. K. Choudhary (Choudhary et al. 2012), *Goniothalamus flagellistylus* Tagane & V. S. Dang (Tagane et al. 2015) and *Vanilla atropogon* Schuit., Aver. & Rybková (Schuiteman et al. 2012), all of which are rare and endemic to Mt. Hon Ba. Therefore further collection efforts around this area are necessary to accurately understand the flora there and to update the conservation status of the species.

## Supplementary Material

XML Treatment for Aporosa tetragona
